# On estimating the number of people with known HIV positive status

**DOI:** 10.1186/s13104-020-04957-y

**Published:** 2020-02-27

**Authors:** Georges Nguefack-Tsague, Serge Clotaire Billong, Ousseni W. Tiemtore, Albert Frank Zeh Meka, Ismael Diallo, Brian Bongwong, Marie Nicole Ngoufack, Ernest Mvilongo, Yemurai Ndowa, Houssey Diallo, Bruno Clary, Koubagnine Takpa, Jean-Baptiste Guiard-Schmid, Leonard Bonono, Jean-Bosco Elat-Nfetam, Jinkou Zhao

**Affiliations:** 1 Biostatistics Unit, Department of Public Health, Faculty of Medicine and Biomedical Sciences, University of Yaoundé I, P.O. Box 8 550, Yaoundé, Cameroon; 2 National AIDS Control Committee, Ministry of Public Health, Yaoundé, Cameroon; 3 Initiatives Conseil International-Santé (ICI-santé), Ouagadougou, Burkina Faso; 4 Hôpital de jour, CHU-Yalgado Ouédraogo, Ouagadougou, Burkina Faso; 5 Centre International de Reference Chantal Biya, Yaoundé, Cameroon; 6 Department of Biochemistry, Faculty of Science, University of Yaoundé 1, Yaoundé, Cameroon; 7 The Global Fund to fight AIDS, Tuberculosis and Malaria, Geneva, Switzerland; 8 UNAIDS, Yaoundé, Cameroon

**Keywords:** 90-90-90 target, Average number of HIV+ tests, HIV cascade, Retesting probability

## Abstract

**Objective:**

In 2014, the Joint United Nations Program on HIV and AIDS (UNAIDS) and partners set the ‘90-90-90 targets’. Many countries are facing the challenge of estimating the first 90. Our objective was to propose an alternative modelling procedure, and to discuss its usefulness for taking into account duplication.

**Results:**

For deduplication, we identified two important ingredients: the probability for an HIV+ person of being re-tested during the period and average number of HIV+ tests. Other adjusted factors included: the false positive probability; the death and emigration probabilities. The uncertainty of the adjusted estimate was assessed using the plausibility bounds and sensitivity analysis. The proposed method was applied to Cameroon for the period 1987–2016. Of the 560,000 people living with HIV estimated from UNAIDS in 2016; 504,000 out to know their status. The model estimates that 380,464 [379,257, 381,674] know their status (75.5%); thus 179,536 who do not know their status should be sought through the intensification of testing. These results were subsequently used for constructing the full 2016 Cameroon HIV cascade for identifying programmatic gap, prioritizing the resources, and guiding the strategies of the 2018–2022 National Strategy Plan and funding request.

## Introduction

In 2014, the Joint United Nations Program on HIV and AIDS (UNAIDS) and partners set the ‘90-90-90 targets’ [1]; aiming by 2020 to (a) diagnose 90% of all HIV positive people (1st 90); (b) provide antiretroviral therapy (ART) for 90% of those diagnosed (2nd 90); and achieve viral suppression for 90% of those treated (3rd 90). UNAIDS Spectrum Software estimates the number of people living with HIV/AIDS (PLWHA). HIV testing is the gateway to HIV prevention, treatment, care and other support services. People’s knowledge of their HIV status through HIV testing services (HTS) is crucial to the success of the HIV response [2]. However, many countries are facing the challenge of estimating the 1st 90 due to lack of directly reported number of individuals diagnosed with HIV.

Estimates of the 1st 90 were generally back calculated using epidemiological models based on data from national HIV testing program or smaller anonymous unlinked seroprevalence surveys, which entails the screening of blood specimens taken for purposes other than HIV testing [1, 3, 4]. Methods include using country reports of the cumulative number of people tested and found HIV positive [5–8].

The method based on the reported number of positive tests, requires unique patient identifiers in case-based surveillance and patient monitoring systems. Such systems are generally not well-established in many settings, where double counting of HIV positive individuals is unavoidable [1]. Double counting will be increasingly important because repeating testing is often encouraged [8, 9]. In fact, WHO [2] recommends that national programs should retest all people newly and previously diagnosed with HIV before they enroll in care and initiate ART; the reasons include ruling out laboratory or transcription error and either ruling in or ruling out seroconversion [10, 11]. WHO [2] also recommends to offer retesting at least annually to people from key population, irrespective of their status. The situation may remain serious, since testing procedures (national algorithm) differ between countries where some require two or more HIV positive tests to confirm diagnosis [3].

Using the number of tests presents a risk of duplication from multiple testing, ultimately leading to overestimation of people with known HIV positive status. Previous methods used mainly subtract from the gross number the false positives, those recorded to have died or permanently migrated [1]. This paper proposes an approach in which both *the probability of being re*-*tested HIV positive* and *the average number of positive tests since 1st positive HIV diagnostic* are taken into account.

## Main text

### Methods

Let ${\text{t}}$ referring the year and ${\text{t}}_{0}$ the base year; ${\text{n}}_{\text{t}}$ the number of HIV positive tests; ${\text{n}}_{\text{tj}}$ the number of HIV positive tests for every testing site; ${\text{K}}_{\text{t}}$ the total number of testing sites. The total number of HIV positive tests during year ${\text{t}}$ is ${\text{n}}_{\text{t}} = \mathop \sum \nolimits_{{{\text{j}} = 1}}^{{{\text{K}}_{\text{t}} }} {\text{n}}_{\text{tj}}$. The cumulative number of HIV positive tests since ${\text{t}}_{0}$ is ${ {\text{N}}}_{t} = \mathop \sum \nolimits_{{{\text{i}} = {{\text{t}}}_{0} }}^{{\text{t}}} {{\text{n}}}_{{\text{i}}} = {{\text{N}}}_{t - 1} + {{\text{n}}}_{{\text{t}}}$. Obviously, ${\text{N}}_{t}$ has duplicates and should thus be adjusted to derive the number of people with known HIV positive status.

Let ${\text{N}}_{\text{t}}^{\text{a}}$ be the adjusted estimate of ${\text{N}}_{t}$ for the period ${\text{t}}_{0}$ to t. A basic adjustment of the estimates is

1 $${\text{N}}_{\text{t}} \left( {1 - {\text{F}}_{t} - {\text{D}}_{t} - {\text{E}}_{t} } \right);$$

where ${\text{F}}_{\text{t}}$ is the false positive probability; ${\text{D}}_{\text{t}}$ the death probability of a tested HIV positive; and ${\text{E}}_{\text{t}}$ the permanent *emigration probability* of a tested HIV positive; i.e. *Out*-*migration of cases.* Within the context of multiple testing, an important ingredient is the number of positive tests realized by an HIV positive individual, which can contribute to inflate the number of PLWHA with known positive status.

Let ${\text{P}}_{\text{t}}$ be the *probability for an HIV*+ *person of being re*-*tested positive during the period*; and ${\text{M}}_{\text{t}}$, the *average number of positive tests*; ${\text{M}}_{\text{t}} \ge 1$. Taking into account the fact that the adjusted estimate (${\text{N}}_{\text{t}}^{\text{a}}$) of the cumulative number of HIV positive tests decreases as ${\text{P}}_{\text{t}}$ and ${\text{M}}_{\text{t}}$ increase, a corrected factor for the basic adjustment in (1) could be $\frac{{{\text{N}}_{{\text{t}}} {{\text{P}}}_{t} }}{{{\text{M}}_{t} }} -{ {\text{N}}}_{{\text{t}}} {{\text{P}}}_{{\text{t}}}$. Similar adjustments have been extensively applied within the context of programmatic mapping to take into account double counting (mobility adjustment) when estimating key populations size [12, 13].

The final estimate is then

2 $$\begin{aligned} {{\text{N}}}_{\text{t}}^{\text{a}} & = {{\text{N}}}_{{\text{t}}} \left( {1 - {{\text{F}}}_{t} - {{\text{D}}}_{t} - {{\text{E}}}_{t} } \right) + \frac{{{{\text{N}}}_{{\text{t}}} {{\text{P}}}_{t} }}{{{\text{M}}_{t} }} - {{\text{N}}}_{\text{t}} {{\text{P}}}_{{\text{t}}} . \\ & = {{\text{N}}}_{{\text{t}}} \left( {1 - {{\text{P}}}_{{\text{t}}} + \frac{{{\text{P}}_{t} }}{{{\text{M}}_{t} }} -{ {\text{F}}}_{t} - {{\text{D}}}_{t} - {{\text{E}}}_{t} } \right) = {{\text{N}}}_{t} \left( {1 - {{\text{F}}}_{t} + \frac{{{\text{P}}_{\text{t}} }}{{{\text{M}}_{\text{t}} }} -{ {\text{P}}}_{\text{t}} - {{\text{D}}}_{{\text{t}}} - {{\text{E}}}_{{\text{t}}} } \right) \\ \end{aligned}$$

The first derivatives of ${\text{N}}_{\text{t}}^{\text{a}}$ with respect to ${\text{P}}_{\text{t}}$ and ${\text{M}}_{\text{t}}$ are $\frac{{1 - {\text{M}}_{\text{t}} }}{{{\text{M}}_{\text{t}} }}$ and $- \frac{{{\text{N}}_{{\text{t}}} {{\text{P}}}_{t} }}{{{\text{M}}_{{\text{t}}}^{2} }}$ respectively, and both are negative.

#### Parameters of the model: data source and methodology

- Probability for an HIV+ person of being re-tested positive during the period ($P_{t}$): ${\text{P}}_{\text{t}}$ can be estimated via a field survey from a nationally representative sample of PLWHA currently on ART.
- Average number of positive tests ($M_{t}$): ${\text{M}}_{\text{t}}$ can also be estimated via a field survey from a nationally representative sample of currently on ART.
- False positive probability ($F_{t}$): It can be obtained from the manufacturers, taking in account the fact that diagnostic test’s characteristics, kits and screening algorithms change over time (period).
- Death probability of a tested HIV positive ($D_{t}$): Death probability can be obtained via a national field survey of representative sample of PLWHA in a prospective cohort study to determine their survival probabilities. It can also be obtained through UNAIDS’s Spectrum Software. Historical data in literature review can be used to determine mortality probability by applying different mortality probabilities according to the period. E.g, taking into account the fact that individual tested HIV+ prior to the introduction of ART had higher probability of dying. Also, the application of different mortality probabilities for PLWHA on ART and those who are not, after scaling up ART. In addition, individual tested HIV+ prior to the introduction of ART had higher probability of dying.
- Permanent emigration probability of a tested HIV positive ($E_{t}$), i.e. out-migration of cases: The permanent emigration probability ($E_{t}$) of a tested HIV positive can be obtained from the National Census Bureau/National Institute of Statistics.

#### Uncertainty estimation

There may be a considerable amount of uncertainty associated with the final adjusted estimate ${\text{N}}_{\text{t}}^{\text{a}}$. To account for that we have calculated uncertainty bounds around each yearly estimate that defines the range within which the true value may situate. A (1 − $\upalpha$)% plausibility bound for the adjusted estimate is [14]:

$$\left[ {\frac{1}{2}X_{{\frac{\alpha }{2}, 2{\text{N}}_{\text{t}}^{\text{a}} }}^{2} ; \quad \frac{1}{2}X_{{1 - \frac{\alpha }{2},2\left( {{\text{N}}_{\text{t}}^{\text{a}} + 1} \right)}}^{2} } \right]$$

where $X_{\beta ,df}^{2}$ the βth percentile of the Chi-square distribution with df as degree of freedom at $\alpha$ level.

An alternative (1 − $\upalpha$)% confidence interval, with normal approximation; nearly equivalent approach is [15]

$$\left[ {{\text{N}}_{\text{t}}^{\text{a}} - Z_{{1 - \frac{\alpha }{2}}} \sqrt {{\text{N}}_{\text{t}}^{\text{a}} } ;\quad {\text{N}}_{\text{t}}^{\text{a}} + Z_{{1 - \frac{\alpha }{2}}} \sqrt {{\text{N}}_{\text{t}}^{\text{a}} } } \right]$$

where $Z_{{1 - \frac{\alpha }{2}}}$ quantile of normal distribution at $\alpha$ level.

These confidence intervals are derived under the assumption that the data follow a Poisson distribution. All analyses were performed in R-3.4.3 (https://www.r-project.org/).

### Results

#### Sensitivity analysis

**Fig. 1:**
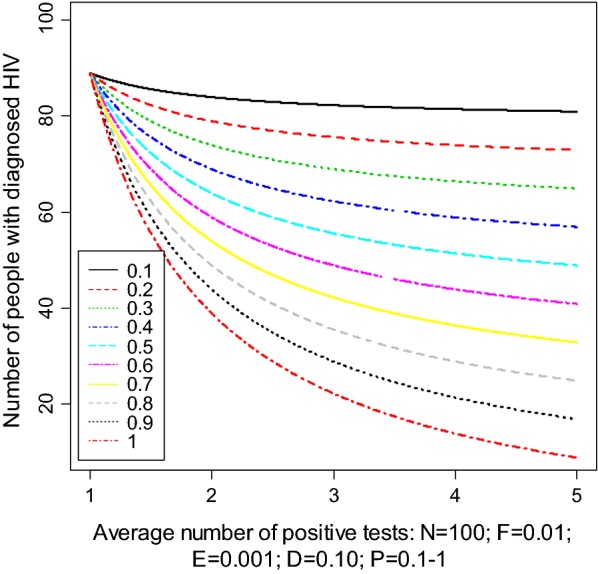
Number of diagnosed HIV as function of average number of HIV+ tests for probabilities of retesting varying between 0.1 to 1

Figure 1 shows the number of diagnosed HIV as function of average number of HIV+ tests at various probabilities of retesting levels; each curve corresponding to a level of probability.^1^

Starting from 100 positive HIV tests, the number of effectively diagnosed HIV positive (${\text{N}}_{\text{t}}^{\text{a}}$) decreases as ${\text{M}}_{\text{t}}$ increases; and also decreases as the probability of retesting (${\text{P}}_{\text{t}} )$ increases. The same trends are observed when varying other parameters such as emigration probabilities, false positive probability, and death probability. The adjusted estimate is then more sensitive to ${\text{M}}_{\text{t}}$ and ${\text{P}}_{\text{t}}$ than other parameters such as ${\text{F}}_{t}$, ${\text{D}}_{t}$ and ${\text{E}}_{t} .$

#### Application of the methods in Cameroon national HIV cascade analysis

The proposed method was used to generate the estimates for the 1st 90 in the 2016 HIV cascade. Table 1 shows the values or range for various model parameters together with their sources.

**Table 1:** Value and source for model parameters

Parameter	Notation	Value	Source
Probability for an HIV+ person of being retested positive during the period	P_t_	0.49	CNLS [16]
Average number of positive tests	M_t_	3.02	CNLS [16]
False positive probability	F_t_	0.01	WHO [27]
Death probability of a tested HIV positive	D_t_	0.042	UNAIDS [28]
Permanent emigration probability of a tested HIV positive	E_t_	0.008	OIM [29]
The cumulative number of HIV positive tests (1987–2016)	N_t_	621,417	CNLS’ reports [30, 31]

**Fig. 2:**
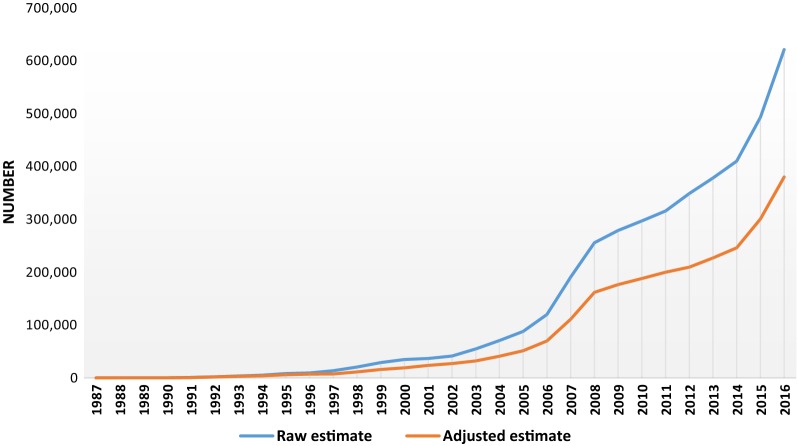
Trends in the number of HIV positive tests (raw estimate) and known HIV positive status (adjusted estimate) from 1987 to 2016 in Cameroon

Figure 2 shows both the raw and the adjusted positive tests from 1987 to 2016. The yearly number of HIV positive tests were obtained from various annual reports of the National AIDS Control Committee from 1987 to 2016 [16, 17]. Of the 560 000 PLWHA estimated from UNAID Spectrum in 2016; 504,000 (0.9 * 560,000) out to know their status. For 621,417 cumulative positive tests in 2016, the model estimates that 380 464 [379,257; 381,674] PLWHA know their status (75.5%; 380,464/504,000). Therefore 179,536 PLWHA who do not know their status should be sought through the intensification of testing.

### Discussion

This paper has proposed a way to take account of duplicates using the number of positive HIV tests in the estimation of the UNAIDS’s 1st 90. In addition to factors such as *the false discovery probability*; the *death probability of a tested HIV*+, and *permanent emigration probability of* a *tested HIV*+; the method included both *the probability of being re*-*tested HIV positive* and *the average number of positive tests since 1st positive HIV diagnostic.*

The estimation of the number of people who know their HIV status remains very difficult. Even in countries with improved monitoring systems such as the United States of America, the number of people living with diagnosed HIV is not available in all states and periods [18]. Moreover, monitoring systems hardly capture the dead as well as those who move that may appear to be lost to follow-up. It is important not only estimating the number of people diagnosed HIV positive, but also considering *s*ome factors favoring the quality and quantity of HIV testing. These include the fact that (a) HIV testing kits are cheap and many countries test for free [19]; (b) repeating testing is often encouraged [8–11, 20]; and (c) reducing discrimination through educational and behavioral program and campaigns [21]. To address the significant shortfall at diagnosis in resource-limited settings, innovative approaches to increase testing coverage should be explored. These include partner testing [22], community-based interventions [23], opt-out testing [22], self-testing [24] and work-based or home-based community testing which was shown to reach up to 80% coverage of testing in high burden countries, but may not be cost-effective in low prevalence countries [25].

Improving the quality of the 1st 90 also involves: (a) putting in place the appropriate strategies to create demand for HIV testing; (b) reaching out and providing targeted services for key populations at increased risk of HIV; (c) putting in place provider-initiated testing and counselling services in health care services (such as tuberculosis, antenatal clinic and sexually transmitted infection services); and (d) encouraging partner testing. The approaches to service delivery should be conducive to increasing uptake of services by the target groups and, eventually, to effective linkage of those diagnosed HIV positive to HIV care and treatment services [2, 26]. The quality of the first 90 can also be improved by using the (a) right strategies/approaches to create demand for HIV testing; (b) testing the right people, and (c) prioritizing the right population groups for HIV testing.

### Conclusions

The application of the method in the Cameroon case showed to be feasible and will hopefully improve estimates of the first 90. The results of this study were subsequently used for constructing the full 2016 Cameroon cascade for identifying programmatic gap, improve targeting of the interventions, to prioritize the resources, guide the strategies of the 2018–2022 National Strategy Plan and the funding request. The proposed model, mostly useful in situations of lack of unique identifier code, can be applied to countries facing similar challenges.

## Limitations

A major challenge in this study was that of accurately selecting the parameter of the proposed model. Some of these parameters can be obtained from nationally representative sample of PLWHA. The proposed method is feasible in the settings where case-based surveillance and patient monitoring systems are not in place. Investments are still needed to improve the case-based surveillance and patient monitoring systems, as the proposed method presents as a temporary solution; routine systems to generate more and better data present as a sustainable solution. The estimate of 1st 90 is justified, when putting in the entire cascade; can be compared with national representative surveys when results are available.

## Data Availability

All data generated or analysed during this study are included in this published article (Fig. 2, Table 1) and Additional file.

## Abbreviations

AIDS: Acquired immunodeficiency syndrome
ART: Antiretroviral therapy
CDC: Centers for Disease Control and Prevention
CNLS: Comité National de Lutte contre le Sida
DHS: Demographic and health surveys
FSW: Female sex worker
HIV: Human immunodeficiency virus
HTS: HIV testing services
IBBS: HIV Integrated Biological and Behavioral Surveillance
MSM: Men who have sex with men
PEPFAR: United States President’s Emergency Fund for AIDS Relief
PLWHA: People living with HIV/AIDS
PMTCT: Prevention of mother to child transmission
STI: Sexually transmitted infections
UNAIDS: Joint United Nations Program on HIV and AIDS
WHO: World Health Organization

## Footnotes

1 Probabilities of retesting ${\text{P}}_{\text{t}}$ varying between 0.1 and 1; emigration probability (${\text{E}}_{t}$) = 0.001, death probability (${\text{D}}_{t}$) = 0.01, and false positive probability (${\text{F}}_{t}$) = 0.01.
